# RETRACTED: Atta et al. Magnetic Ionic Liquid Nanocatalyst to Improve Mechanical and Thermal Properties of Epoxy Nanocomposites. *Nanomaterials* 2020, *10*, 2325

**DOI:** 10.3390/nano16050293

**Published:** 2026-02-26

**Authors:** Ayman M. Atta, Hamad A. Al-Lohedan, Ahmed M. Tawfeek, Nourah I. Sabeela

**Affiliations:** Department of Chemistry, College of Science, King Saud University, Riyadh 11451, Saudi Arabia; hlohedan@ksu.edu.sa (H.A.A.-L.); a.tawfik@ksu.edu.sa (A.M.T.); noonah-37@hotmail.com (N.I.S.)

The journal retracts the article entitled “Magnetic Ionic Liquid Nanocatalyst to Improve Mechanical and Thermal Properties of Epoxy Nanocomposites” [1], cited above.

Following publication, concerns were brought to the attention of the Editorial Office regarding the integrity and accuracy of the images and data presented in the publication [1].

In accordance with the journal’s standard procedures, the Editorial Office and Editorial Board conducted an investigation that identified multiple indications of inappropriate image editing and panel duplication between this article and a significant number of previously published works by the same authorship group. These concerns relate to the images and data presented in Figures 5, 11, and 12. Following further discussions with the authors, the Editorial Board was unable to verify the integrity of the figures mentioned from the explanations and supplementary material provided. Consequently, the Editorial Board has lost confidence in the reliability of the overall findings and has decided to retract this publication [1], as per MDPI’s retraction policy.

This retraction was approved by the Editorial Board Member and Editor-in-Chief of the journal.

The authors have been informed of this retraction but did not provide a comment on this decision.
